# Occupational risk of COVID-19 in the first versus second epidemic wave in Norway, 2020

**DOI:** 10.2807/1560-7917.ES.2021.26.40.2001875

**Published:** 2021-10-07

**Authors:** Karin Magnusson, Karin Nygård, Fredrik Methi, Line Vold, Kjetil Telle

**Affiliations:** 1 Norwegian Institute of Public Health, Cluster for Health Services Research, Oslo, Norway; 2 Lund University, Faculty of Medicine, Department of Clinical Sciences Lund, Orthopaedics, Clinical Epidemiology Unit, Lund, Sweden; 3 Norwegian Institute of Public Health, Department of Infection Control and Preparedness, Oslo, Norway

**Keywords:** COVID-19, occupational risk, pandemic policy

## Abstract

**Background:**

The occupational risk of COVID-19 may be different in the first versus second epidemic wave.

**Aim:**

To study whether employees in occupations that typically entail close contact with others were at higher risk of SARS-CoV-2 infection and COVID-19-related hospitalisation during the first and second epidemic wave before and after 18 July 2020, in Norway.

**Methods:**

We included individuals in occupations working with patients, children, students, or customers using Standard Classification of Occupations (ISCO-08) codes. We compared residents (3,559,694 on 1 January 2020) in such occupations aged 20–70 years (mean: 44.1; standard deviation: 14.3 years; 51% men) to age-matched individuals in other professions using logistic regression adjusted for age, sex, birth country and marital status.

**Results:**

Nurses, physicians, dentists and physiotherapists had 2–3.5 times the odds of COVID-19 during the first wave when compared with others of working age. In the second wave, bartenders, waiters, food counter attendants, transport conductors, travel stewards, childcare workers, preschool and primary school teachers had ca 1.25–2 times the odds of infection. Bus, tram and taxi drivers had an increased odds of infection in both waves (odds ratio: 1.2–2.1). Occupation was of limited relevance for the odds of severe infection, here studied as hospitalisation with the disease.

**Conclusion:**

Our findings from the entire Norwegian population may be of relevance to national and regional authorities in handling the epidemic. Also, we provide a knowledge foundation for more targeted future studies of lockdowns and disease control measures.

## Introduction

The coronavirus disease 2019 (COVID-19) emerged in late 2019 in China and has, as of September 2020, resulted in over 29,000,000 infections and over 900,000 deaths globally [1]. The first cases with confirmed COVID-19 in the Nordic countries were probably imported when residents visited bars and restaurants in Austria and Italy during winter holidays in February 2020 [1]. Later, lockdown and restrictions in the retail, catering and tourism industries are believed to have dramatically reduced the spread of the virus. The closure of schools and preschools are assumed to have had a smaller effect [2-5]. However, to what extent occupational settings that normally imply close contact with patients, children, students or customers contribute to the spread of COVID-19 and its severity is currently unknown.

Only a few studies have been published on the occupational risk of COVID-19, and these have mainly focused on disease severity or mortality. The first reports of occupational risk of COVID-19 were from Singapore in early February 2020, which showed that 25 locally transmitted cases were employed in tourism and trading [6]. A later British study reported that essential workers such as personal service occupations and plant and machine operators had a higher risk of severe COVID-19 than non-essential workers, who are believed to work more from a home-office setting [7]. In England, Wales and Sweden, occupations in sales and retail, transport (bus/taxi drivers) and catering (chefs) had elevated mortality rates of COVID-19, whereas teachers had lower mortality rates [8,9].

An overview of the pattern of COVID-19 and accompanying utilisation of healthcare services in individuals employed in a wide range of occupations is currently lacking. Improved knowledge of occupational risk would greatly contribute to informing authorities on whether certain activities in these sectors should be ‘locked down’ in attempts to limit the spread of the severe acute respiratory syndrome coronavirus 2 (SARS-CoV-2) with its severe outcomes. In Norway, there was a lockdown as well as closure of schools and childcare centres during spring 2020. Restrictions were eased in the summer of 2020. However, as transmission increased during fall 2020, several restrictions were re-implemented, including bans on serving alcohol from November 2020.

At the time of writing, most European countries including Norway experienced two epidemic waves [10], one during spring 2020, and one during fall 2020, which because of the novelty of SARS-CoV-2 and restrictions undertaken may be associated with different occupational risks. As an example, healthcare workers may have been particularly exposed to transmission in the beginning of the COVID-19 pandemic, as few preventive measures were implemented. Thus, we aimed to study the occupational risk of COVID-19 and its severity (hospitalisations) for all individuals in the Norwegian population of working age (20–70 years) employed in health professions, education and teaching, retail, catering, travel, tourism and recreation industries during the two epidemic waves in Norway. 

## Methods

### Study design

We used individual-level data from the BEREDT C19 register to form an observational prospective cohort study covering the entire Norwegian population during the period from 26 February to 18 December 2020. BEREDT C19 is a newly developed emergency preparedness register aimed at providing rapid knowledge of the spread of SARS-CoV-2, as well as how the spread and measures to limit the spread affect the population's health, use of healthcare services and health-related behaviours [11].

### Data sources

BEREDT C19 consists of electronic patient records from all hospitals in Norway (Norwegian Patient Registry), data from the Norwegian Surveillance System for Communicable Diseases (MSIS), The Norwegian Population Registry and the Employer–Employee register, which are merged on the unique personal identification number that is assigned to every Norwegian resident at birth or upon immigration. On account of BEREDT C19, our study comprises all Norwegian residents including immigrants. Data were updated daily (except for the Employer–Employee register, which was updated on 25 August 2020) and span the entire year of 2020. BEREDT C19 includes data on residents in Norway who have tested positive for SARS-CoV-2, with dates of testing and diagnosis, which are legally required to be reported to MSIS from all laboratories and physicians. The register also includes the date(s) of any hospitalisation, with complete diagnostic codes from 1 January 2020. 

Occupation is reported in the Employer–Employee register with standard classification of occupations codes, as described at Statistics Norway for all residents in Norway [12]. Thus, in the current study, we were able to include all living Norwegian residents in the working age group defined in this study as between 20–70 years on 1 January 2020. Non-residents (like tourists, temporary workers and asylum applicants) were excluded. 

### Occupation

Occupation was registered with a seven-digit code in the Employer–Employee register according to the Standard Classification of Occupation (STYRK-98) [12]. To allow for international comparisons, we used a conversion table to align the classification with the International Standard Classification of Occupations (ISCO-08) using four-digit codes, i.e. corresponding to the Norwegian STYRK-08 [12,13]. We selected common occupations with a total number of employees of 1,000 or more and the number of contracted weekly work time of 1 h or more for a reference week at the beginning of the pandemic (week 10). 

The occupations investigated in this study were chosen as they usually imply direct contact with other people in different user groups, and were classified into occupations within health, teaching, retail, catering, tourism and travel, recreation and beauty (Table 1). Individuals not registered with any of the STYRK codes were classified as ‘everyone else in their working age (20–70 years)’ and included individuals with other occupations with an assumable low degree of contact with patients, children, students or customers. This category also included individuals in the population register who had a missing value on the employment code for an unknown reason (unemployed individuals, non-employees, individuals on disability pensions, those seeking work, freelancing and self-employed individuals and students).

**Table 1:** Occupations in industries having direct contact with patients, children, students or customers, Norway, 2020

Occupation categories	Code^a^
**Health occupations**	
Physicians	2211/2212
Nurses	2221/2223
Dentists	2261
Physiotherapists	2264
**Teaching occupations **	
Primary school teachers	2341
Early childhood educators/preschool teachers	2342
Childcare workers	5311
Secondary education teachers	2330
University and higher education teachers	2310
**Retail occupations **	
Shop sales assistants	5223
Cleaners	9112
**Catering occupations **	
Waiters	5131
Bartenders	5132
Food service counter attendant	5246
**Tourism and travel occupations **	
Hotel receptionists	4224
Travel guides	5113
Travel attendants and travel stewards	5111
Transport conductors	5112
Bus and tram drivers	8331
Car, taxi and van drivers	8322
**Recreation and beauty occupations **	
Fitness and recreation instructors and programme leaders	3424
Hair dressers	5141

^a^ According to the International Standard for Classification of Occupation (ISCO-08 / STYRK-08).

### Outcomes

We studied two outcomes: (i) COVID-19, which was defined as either having a confirmed positive RT-PCR test for SARS-CoV-2 and/or by having the ICD-10 diagnostic code U07.1 of confirmed COVID-19 and (ii) hospitalisation for at least 24 h with confirmed COVID-19 [14]. Test criteria for COVID-19 initially included having severe disease, being in a risk group or being health personnel; this later changed to include everyone with symptoms (e.g. cough or fever) or having been in contact with individuals with confirmed COVID-19. We studied our outcomes for two periods, before and after 18 July 2020. On this date, the number of incident infections were low and remained low through July before slowly rising again in the beginning of August [1-5]. We refer to the two periods as the first wave (26 February–17 July 2020) and the second wave (18 July–20 October 2020).

### Statistical analyses

We estimated the total number of confirmed COVID-19 cases per 1,000 employed individuals for the two epidemic waves for each of the occupation groups. We next assessed the crude association between each of the exposure occupation groups (i.e. a categorical variable including the 22 categories, one for each occupation) and the outcome ‘confirmed COVID-19’ (yes/no) using logistic regression separately for each of the waves and reporting odds ratios (OR). Then, we assumed that several potential covariates may confound the association between occupation and wave-specific COVID-19 outcome, so we adjusted for the following covariates in three multivariate logistic regression models: (i) age and sex, (ii) age, sex, country of birth and mother’s country of birth (because transmission has been reported to be particularly high in immigrant groups [15]) and (iii) age, sex, country of birth, mother’s country of birth and marital status. Given the large number of observations, we implemented the covariates as categorical variables (five age categories: 20–29, 30–39, 40–49, 50–59, 60–70 years; seven categories for one’s own and the maternal country of birth (in separate variables): born in Norway, rest of Europe, Asia, Africa, Latin America, North America or Oceania, or unknown). We set ‘everyone else in their working age (20–70 years)’ to be the reference category in all analyses. Finally, we repeated the analyses using hospitalisation with COVID-19 as outcome with additional adjustment for the number of comorbidities (none, one, two, or three or more comorbidities), however, due to a low number of hospitalisations for several occupation groups, we did not separate these analyses on the first and second wave. The statistical software used was STATA MP (version 16, STATACorp, College Station, Texas, United States).

### Ethical statement

Institutional board review was conducted, and the Ethics Committee of South-East Norway confirmed on 4 June 2020 (#153204) that external ethical board review was not required.

## Results

**Figure 1:**
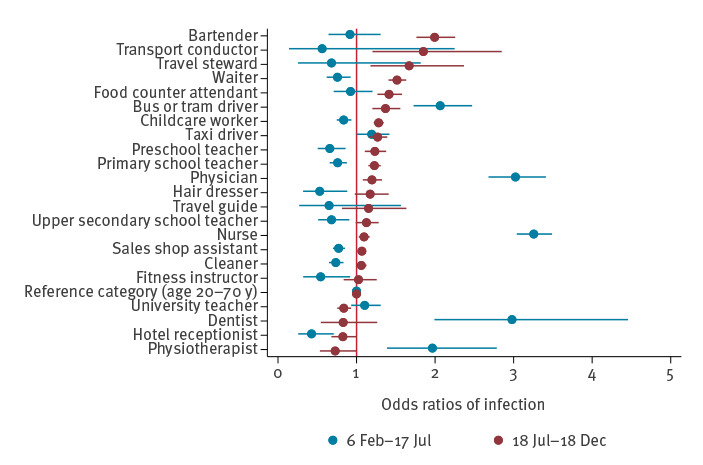
Odds ratios of COVID-19 by occupation during the first (26 February–17 July) and second (18 July–18 December) wave, adjusted for age, sex, own and maternal country of birth and marital status, Norway, 2020 (n = 3,579,608)

We studied in total 3,559,694 individuals aged 20–70 years living in Norway on 1 January 2020 (4,715,542 registered employment contracts), with a mean age of 44.1 years (standard deviation: 14.3) and consisting of 51% men. Of these, 74.2% had Norway as the birth country (50% of those not born in Norway were born in another European country) and 24.4% were not employed or not registered with any occupation. By 18 December 2020, a total of 30,003 (0.8%) had contracted COVID-19, of which 1,550 (5.2%) had been hospitalised with COVID-19. The proportions with COVID-19 and related hospitalisation per occupation are reported (Table 2). There were considerable differences in occupation-wise incident cases in the first versus the second epidemic wave (Table 2 and Figure 1).

**Table 2:** Absolute numbers of COVID-19 and incidence proportions for infections and hospitalisations per occupation per 1,000 infected employees, Norway, 26 February–18 December 2020 (n = 3,579,608)

**Occupation categories**	**Total period ** **26 Feb–18 Dec**					**First wave** **26 Feb–17 Jul**		**Second wave ** **18 Jul–18 Dec**	
	**Total**	**Infections**		**Hospitalisations^a^**		**Infections**		**Infections**	
	**n**	**n**	**%**	**n**	**%**	**n**	**%**	**n**	**%**
Individuals of working age, 20–70 years	3,579,608	31,675	9	1,469	46	7,497	2	24,184	7
Health occupations									
Physicians	152,151	2,032	13	43	21	990	7	1,042	7
Nurses	45,320	665	15	24	36	281	6	384	8
Dentists	3,845	46	12	11	239	24	6	22	5
Physiotherapists	NA				14	NA	3	NA	4
Teaching occupations									
Primary school teacher	130,483	1,204	9	34	28	192	1	1,012	8
Early childhood educators	45,144	387	9	14	36	55	1	332	7
Childcare workers	174,118	2,042	12	49	24	333	2	1,709	10
Secondary education teachers	38,284	281	7	11	39	47	1	234	6
University and higher education teachers	60,807	496	8	19	38	137	2	359	6
Retail occupations									
Shop sales assistant	238,689	2,605	11	59	23	425	2	2,180	9
Cleaners	109,202	1,418	13	62	44	264	2	1,154	10
Catering occupations									
Waiters	47,422	845	18	21	25	99	2	746	16
Bartenders	15,290	293	19	9	31	31	2	262	17
Food service counter attendant	18,809	385	20	14	36	56	3	329	17
Tourism and travel occupations^b^									
Hotel receptionists	NA		8	NA	26	NA	1	NA	7
Travel guides	NA		9	NA	0	NA	1	NA	8
Travel attendants and travel stewards	NA		12	NA	0	NA	2	NA	11
Transport conductors	NA		12	NA	87	NA	1	NA	11
Bus and tram drivers	21,157	364	17	34	93	125	6	239	11
Car, taxi and van drivers	35,213	609	17	46	76	135	4	474	13
Recreation and beauty occupations^b^									
Fitness and recreation instructors and programme leaders	NA				0	NA	1	NA	7
Hair dressers	NA				31	NA	1	NA	8

COVID-19: coronavirus disease; NA: data not available.

^a^ The percentages here are based on per cent hospitalised/infected, not on the total.

^b^ If an occupation had one or more cells with n < 5, we refrained from reporting any absolute numbers for that occupation.

COVID-19: coronavirus disease.

The reference category was all other individuals of working age (20–70 years), denoted by the vertical red line (odds ratio = 1). Solid circles represent odds ratios for each occupation and corresponding bars represent the 95% confidence intervals.

### Outcome of COVID-19 during the first wave, 26 February–17 July 2020

**Figure 2:**
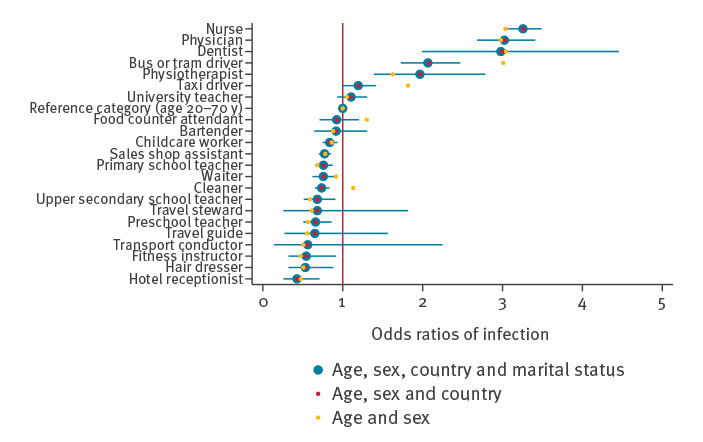
Odds ratios of COVID-19 during the first wave, adjusted for age, sex, own and maternal country of birth and marital status, Norway, 26 February–17 July 2020 (n = 3,579,608)

Individuals employed as nurses, physicians, dentists, physiotherapists, bus, tram or taxi drivers had ca 1.5–3.0 times the odds of confirmed COVID-19 during the first wave when compared with everyone of working age (Figure 2). In contrast, teachers of children and students of any age, childcare workers, as well as bartenders, waiters, sales shop assistants, cleaners, fitness instructors, hair dressers, hotel receptionists, travel guides and transport conductors had no increased risk, or even a reduced risk of confirmed COVID-19 when compared with others of working age (Figure 2). Generally, point estimates were closer to an OR of 1 in analyses adjusted for age, sex and country of birth when compared with crude analyses (Figure 2).

COVID-19: coronavirus disease.

The reference category was all other individuals of working age (20–70 years), denoted by the vertical red line (odds ratio = 1). Solid circles represent odds ratios for each occupation and corresponding bars represent the 95% confidence intervals.

### Outcome of COVID-19 during the second wave, 18 July–18 December 2020

**Figure 3:**
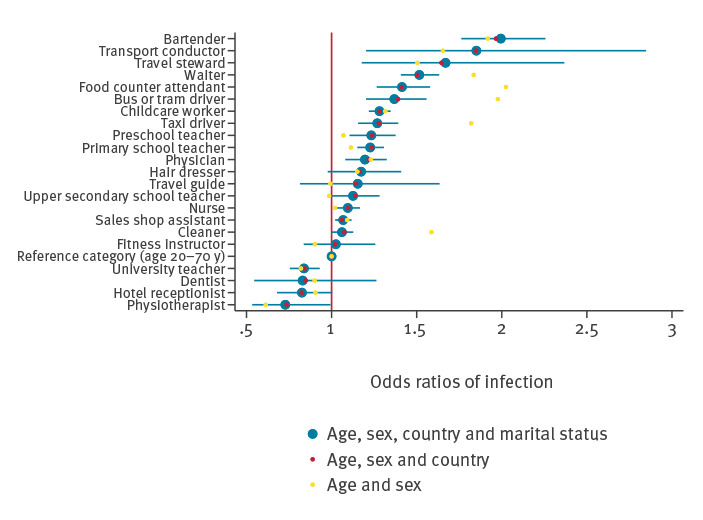
Odds ratios of COVID-19 during the second wave adjusted for age, sex, own and maternal country of birth and marital status, Norway, 18 July–18 December 2020 (n = 3,579,608)

The pattern of occupational risk of confirmed COVID-19 was different for the second epidemic wave than for the first wave. In the second wave, bartenders, transport conductors, travel stewards, waiters and food service counter attendants had ca 1.5–2 times greater odds of COVID-19 when compared with everyone of working age (Figure 3). A range of occupations had moderately increased odds (OR: ca 1.1–1.5): bus and tram drivers, childcare workers, taxi drivers, teachers of children and at any age, physicians, hair dressers, nurses, sales shop assistants, and cleaners when compared with others of working age (Figure 3). University teachers, dentists, hotel receptionists and physiotherapists had no increased odds (Figure 3). Again, point estimates were closer to an OR of 1 in analyses adjusted for age, sex, one’s own and maternal country of birth, as well as marital status when compared with crude analyses (Figure 3).

COVID-19: coronavirus disease.

The reference category was all other individuals of working age (20–70 years), denoted by the vertical red line (odds ratio = 1). Solid circles represent odds ratios for each occupation and corresponding bars represent the 95% confidence intervals.

### Outcome of hospitalisation with COVID-19

**Figure 4:**
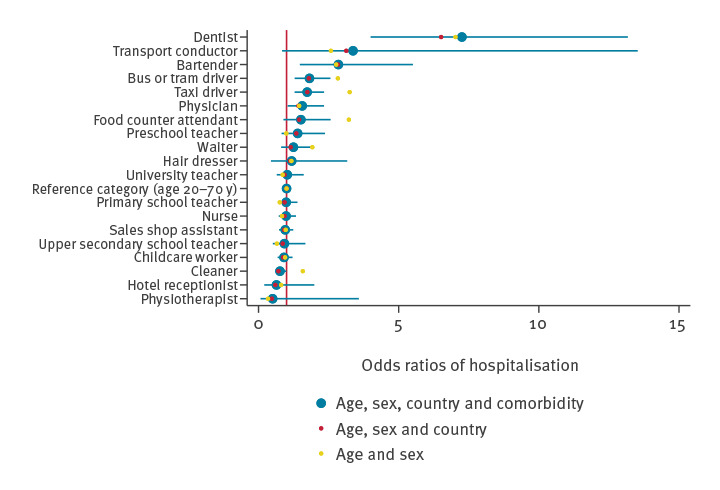
Odds ratios of COVID-19-related hospitalisation during the first and second waves adjusted for age, sex, own and maternal country of birth and comorbidities, Norway, 26 February–18 December 2020 (n = 3,579,608)

None of the included occupations had a particularly increased risk of severe COVID-19, indicated by hospitalisation, when compared with all infected individuals of working age (Figure 4), apart from dentists, who had an OR of ca 7 (95% CI: 2–18) times greater; preschool teachers, childcare workers and taxi, bus and tram drivers had an OR of ca 1–2 times greater. However, for several occupations, no hospitalisations were observed, confidence intervals were wide and all analyses should be interpreted with care because of the small number of COVID-19 hospitalisations (Figure 4).

COVID-19: coronavirus disease.

The reference category was all other individuals of working age (20–70 years), denoted by the vertical red line (odds ratio = 1). Solid circles represent odds ratios for each occupation and corresponding bars represent the 95% confidence intervals.

## Discussion

By studying the entire Norwegian population, we were able to identify a different pattern of occupational risk of COVID-19 for the first and the second epidemic wave. Health personnel (nurses, physicians, dentists and physiotherapists) had 2–3.5 times greater odds of contracting COVID-19 during the first wave when compared with all individuals of working age. In the second wave, bartenders, waiters, food counter attendants, transport conductors, travel stewards, childcare workers, preschool and primary school teachers had ca 1.1–2 times greater odds of COVID-19. Bus, tram and taxi drivers had an increased odds of contracting COVID-19 in both waves (OR ca 1.2–2.1). However, we found indications that occupation may be of limited relevance for the risk of severe COVID-19 and the need for hospitalisation.

This report is the first to our knowledge to show the risks of contracting COVID-19 for specific occupations for the entire working population and for everyone diagnosed. Existing reports have considered these associations in smaller populations, have used broader categories of occupations and/or have considered only severe, hospital-confirmed COVID-19 or mortality [6-9]. Here, we studied all individuals of working age with a positive RT-PCR test for SARS-CoV-2 in Norway in addition to all hospital-confirmed COVID-19 and all hospitalisations with COVID-19. In order to examine different occupations, we used the internationally well-known ISCO-codes with four digits, and applied simple logistic regression models, which will make analyses easily reproducible and comparable when repeated in other countries or in other study samples. In that regard, by making use of all available data for the entire Norwegian population, our findings are representative for other countries that give equal access to healthcare, including COVID-19 testing to all inhabitants. 

We confirm results from a study from the Swedish public health agency, which reports more transmission among waiters and taxi, bus and tram drivers [16]. However, in contrast to the Swedish study, we report a higher risk of transmission among teachers during the fall of 2020 than what has been observed in Sweden during the spring of 2020 [16]. We believe the two periods across the two countries are comparable, provided that schools were largely closed in Norway during the spring of 2020 but were open in Norway during fall 2020 and in Sweden during spring 2020. Potential explanations for the differing findings are unknown, but may be related to different samples and comparison groups. For any comparison of our findings to those in other countries, it should be noted that transmission of COVID-19 has been relatively low in Norway when compared with other countries.

Considering that workers may both become infected through their occupation but may also spread SARS-CoV-2 to their patients, children, students or customers, our findings may have implications for pandemic policy. They do not support that teachers were at higher risk of infection in the first epidemic wave, when many schools were closed in Norway. In the second wave, however, when most schools for children closed only in case of detected infections, preschool and primary school teachers did have moderately elevated infection rates. Bartenders, waiters, travel stewards, bus, tram and taxi drivers had a higher risk of infection than other occupation groups in the first and/or second epidemic wave. They also typically have contact with many different people in their work, possibly exposing their customers or clients while at work if they are not aware that they are infected. These findings may be of relevance for the future considerations of restrictions and/or the use of face masks in certain occupational settings. Our findings also raise important hypotheses for future research. As an example, although we had few cases and considerable uncertainty in our analyses of COVID-19-related hospitalisation, our results may indicate that dentists are at increased risk of severe COVID-19, raising important new hypotheses regarding the relevance of viral load or infectious doses in causing severe disease.

Except for our analyses of hospitalisation, we chose to divide our analyses in two periods, encompassing the first and second epidemic waves, using an arbitrary cut-off for a period when transmission was low (18 July, i.e. dividing our total study period into two equal 5-month periods) [10]. Along this line, an important potential explanation for the differing findings in the first and second wave may be differences in test criteria in Norway throughout the year, which changed from including only those with severe disease, at risk and/or health personnel before the summer, to include everyone with mild symptoms and/or those who had been in contact with individuals having confirmed COVID-19 after the summer. These differences in test criteria may also explain why health personnel were at increased risk during the first wave but not the second wave. However, it is also possible that health personnel have implemented better infection control measures, resulting in fewer healthcare workers (e.g. nurses, dentists, and others) being infected as the pandemic progressed. Future research should further detail the association between type of health/medical occupation and infection risk in different time periods when different infection control measures were implemented i.e. to distinguish between occupations in specialist and primary care or nursing and elderly homes [17].

Another issue of importance to the interpretation of our findings is that 24% of the working age population could not be categorised using available registry data i.e. this group ranges from students and freelance workers to those who are unemployed or have a disability pension. As an example, the individuals infected during the second wave were younger and likely consisted of more students when compared with individuals infected in the first wave [1-5]. This may be explained by younger adults being less adherent to restrictions and preventive measures than the older people as the pandemic progressed in the second wave. Students, typically aged 20–25 years, may more often have no occupation and/or more often have part-time work as bartenders, waiters, food counter attendants, childcare workers and sales shop assistant than those aged 30 years and older, potentially explaining our results. Unemployed people might also be on disability pensions because of poor health and at greater risk of severe COVID-19, potentially explaining why our findings indicate limited occupational risk of hospitalisation with COVID-19. In total, 12% of non-elderly adults (under 67 years) in Norway are on disability pensions. Also, the proportion of fully or partially retired individuals increases from 0% to ca 95% between the ages of 60 and 70 [18], and they may be exposed to a minimal or considerable occupational risk.

Some important limitations should be mentioned. First, we cannot exclude that other factors than the occupation in question explain infection and hospitalisation risks in our study. As an example, individuals in full-employment may be at greater risk of COVID-19 than individuals in part-time employment. Also, we cannot be sure we have sufficiently adjusted for other risk factors related to e.g. country of birth, residential area, risky behaviour and health literacy, which may be of particular relevance to our analyses of hospitalisation [5]. Further, it is possible that employees working and living close together in small spaces (more typical for big cities) may be infected by each other rather than by the patients, children, students or customers they meet [18]. Indeed, point estimates and their 95% confidence intervals were lower in adjusted analyses compared with crude analyses, suggesting that occupation and our outcomes are partly explained by sociodemographic factors. Thus, we cannot distinguish transmissions between colleagues from transmissions between users and employees [19,20]. Finally, we converted the Norwegian occupation classification STYRK-98 to STYRK-08/ISCO-08 and some of the occupations (0.3%) were lost as they did not convert to the international system [12,13]. The reference category was calculated using STYRK-98.

In conclusion, we show that nurses, physicians, dentists and physiotherapists had the highest risk of confirmed COVID-19 during the first wave in Norway, which shifted to bartenders, waiters, food counter attendants, transport conductors, travel stewards and childcare workers, preschool and primary school teachers during the second wave. Bus, tram and taxi drivers had a high risk of COVID-19 in both waves. Our findings may be of relevance to increase the understanding of risk and transmission settings for COVID-19 in order to contribute to more targeted measures to decrease transmission of COVID-19 in public settings.
