# Erratum for Euro Surveill. 2021;26(40)

**DOI:** 10.2807/1560-7917.ES.2021.26.41.211014e1

**Published:** 2021-10-14

**Authors:** Eurosurveillance editorial team

**Affiliations:** 1 European Centre for Disease Prevention and Control (ECDC), Stockholm, Sweden

In the article ‘Occupational risk of COVID-19 in the first vs second epidemic wave in Norway, 2020’ by Magnusson et al. published on 7 October 2021, the following corrections were made to Table 2: the % in the column headings were corrected to ‰, and the variables ‘Nurses’ and ‘Physicians’ in the left column were in the incorrect order and have been updated. In addition, the date of 20 October 2020 was amended to 18 December 2020 in the Methods. The errors were corrected on 14 October 2021. We apologise for any inconvenience this may have caused.

